# Gingival Depigmentation Using Diode 980 nm and Erbium-YAG 2940 nm Lasers: A Split-Mouth Clinical Comparative Study

**DOI:** 10.1155/2021/9424793

**Published:** 2021-12-28

**Authors:** Zaid Kamel Jnaid Harb, Walid El-Sayed, Jumma Alkhabuli

**Affiliations:** ^1^Royal Clinic, Corniche Alqawasim, Ras Alkhaimah, UAE; ^2^College of Dental Medicine, Gulf Medical University, Ajman, UAE; ^3^College of Dentistry, Suez Canal University, Ismailia, Egypt; ^4^Oral Medicine, College of Dental Medicine, QU Health, Qatar University, Doha, Qatar

## Abstract

**Background:**

Gingival hyperpigmentation, “black gum,” refers to black discrete single or multiple pigments on the gingiva. Several factors may play a role in gingival hyperpigmentation ranging from physiologic pigmentation to manifestations of systemic diseases. Several techniques have been used for gingival depigmentation to lighten its color.

**Methods:**

Fifteen patients exhibiting nonsmoking melanin hyperpigmentation, with the mean age of 28.6 ± 7.8 years, were recruited. The facial gingiva of the anterior teeth and premolars of each jaw was divided into two halves. The right or left side of each jaw quadrant randomly received either diode laser operating at 980 nm wavelength or erbium-YAG laser at 2940 nm. Parameters such as degree of gingival depigmentation, bleeding, pain, patient satisfaction, and wound healing were assessed and compared between the two techniques. The subjects were followed up to six months for melanin pigmentation recurrence.

**Results:**

Both techniques were efficient for gingival depigmentation. Nevertheless, bleeding during surgery was statistically higher for Er:YAG laser technique as compared to diode laser. Wound healing showed statistically nonsignificant differences between the two lasers, although Er:YAG seems to give better outcomes than the diode. The patients were satisfied with both laser techniques during and after gingival depigmentation. However, the pain score was higher for Er:YAG laser than for diode laser.

**Conclusion:**

This study demonstrated that both lasers' techniques are efficient for gingival depigmentation. However, diode laser seems to show less painful experience and relatively better bleeding control.

## 1. Introduction

Recently, cosmetic dentistry has attracted more attention among the public as it can empower the self-confidence of individuals. A perfect smile is not only influenced by the shape and position of teeth or lips but also influenced by the outlook of the gingival tissues [1]. The normal color of healthy gingiva is coral or salmon, although it may vary from pale pink to dark bluish-purple [2]. This physiological variation is controlled and determined by several factors including the degree of vascularization, the thickness of the gingival epithelium, keratinization of the stratum layer, and the amount of melanin pigments within the epithelium [2].

Gingival hyperpigmentation (GHP), “black gum,” is considered one of the most important elements affecting the appearance of an individual's smile [3]. GHP is due to the excessive release of melanin pigments in the gingival epithelium [4]. During early embryogenesis, melanocyte-progenitor cells migrate from the neural crest and rest in the basal layer of the skin epidermis and epithelium of mucous membranes, where they undergo maturation as melanocytes. The ratio of melanocytes to keratinocytes in the basal layer ranges from 1 : 10 to 1 : 15, although this may vary at different growth and development stages [5].

GHP is a multifactorial problem caused by various lesions and conditions. Physiologic or racial pigmentation is not uncommon among dark-skinned individuals. It may be caused by other endogenous and exogenous factors associated with systemic diseases, genetic factors, or smoking [5]. The prevalence of physiological or smoking-related oral pigmentation can reach up to 89% among populations [6]. Clinically, physiological GHP exhibits asymptomatic, multiple or single, well-defined, light-to-dark brown macules of variable sizes that may affect any part of the oral mucosa, particularly gingiva [5]. Pathological oral hyperpigmentation is caused by excessive melanin pigmentation associated with systemic diseases, such as Addison disease, neurofibromatosis, or Peutz-Jeghers syndrome [7]. Heavy smoking has a high tendency towards pigmentation (smoker's melanosis). Furthermore, drugs, such as antimalarials, tetracyclines, ketoconazole, and zidovudine also are linked with melanin pigmentation [7].

Gingival depigmentation (GD) is a surgical procedure used to remove or reduce excessive pigmentation applying various treatment approaches including scalpel surgery, cryosurgery, gingivectomy, free gingival graft, gingival abrasion, radiosurgery, and recently numerous types of lasers [8]. The procedure is aiming to target melanin pigments and melanin-producing cells to lighten the color of the gingiva. The selection of a GD technique is mainly based on clinical experience and patient preferences [9–11].

Lasers have been widely used in medicine and dentistry for various treatment modalities since 1970 [12]. The use of various lasers has been reported as reliable, safe, and efficient causing minimal bleeding and postoperative pain. In addition, it can precisely cut, ablate, and reshape gingival tissues [13].

The main objective of this study was to compare the efficacy of two types of lasers, namely, diode laser 980 nm and erbium-YAG laser 2940 nm for GD and to assess several outcome factors including bleeding, wound healing, postoperative pain, and pigmentation recurrence. We hypothesise that there is no significant difference between diode laser 980 nm and erbium-YAG laser 2940 nm for gingival depigmentation.

## 2. Material and Methods

The study obtained the approval of the Research and Ethics Committee (UNIGE-REC18-2017). A convenience sampling method was applied for sample size estimation. All patients attending the dental center complaining of gum pigmentation and seeking gum lightening during the period extending from January to June 2017 were considered in the study. Only 15 patients were found to meet the strict inclusion/exclusion criteria. All subjects provided written consent to receive laser ablation treatment. Only physiological GHP cases were included. The subjects' details were recorded and kept confidential. The technical procedure was explained to the participants and signed informed consent. Neither topical nor local anesthesia was used in the procedure. Oral prophylaxis including supra- and subgingival scaling was performed before laser therapy, and the participants were asked to follow strict oral hygiene instructions. Chlorhexidine gluconate mouthwash was prescribed for 7 days to control dental plaque. Analgesics were prescribed to control postoperative pain if any. The postoperative instructions were given to subjects and informed of the potential complications.

Each patient received laser treatment for the 4 quadrants of the mouth: 2 by erbium-YAG and 2 by diode laser selected randomly.

The GD procedure was performed using either erbium-YAG or diode laser. Assessment of the pain, healing patterns, bleeding, and the efficiency of GD was performed by close monitoring of the participants through periodic visits over 6 months to evaluate any pigmentation recurrence.

Er:YAG laser (2940 nm) wavelength (LAMPDA Doctor Smile; Italy, Class 4 laser product) was used in all patients. The laser parameters and specifications are summarized in Table 1. In general, Er:YAG laser radiation was used with emission mode free running pulse, 2 watts, 20 Hz, 1.5 ml/min H20, and the tip in fiber-optic handpiece was used with 800 microns, noncontact mode, and 1 mm distance from the tissue for 600 seconds.

Diode laser (980 nm) wavelength (LAMPDA Doctor Smile; Italy, Class 4 laser product) was used in all patients. The laser parameters and specifications are summarized in Table 1. In summary, a 400*μ* tip in a fiber-optic handpiece was initiated (8 times, per setting) using a power range of 0.80–1.10 watts with angulations of the tip 12.7 degrees. Continuous-wave, contact mode parameters were used during the procedure in a coronoapical direction movement of the pigmented areas for a range of 240–600 seconds. A surgical aspirator was used to cool the operative site. Sterile gauze soaked in saline was used to remove the char formed over the surface of the surgical areas. Extreme care was taken by a thorough examination to ensure the removal of all epithelial pigmented areas.

**Figure 1:**
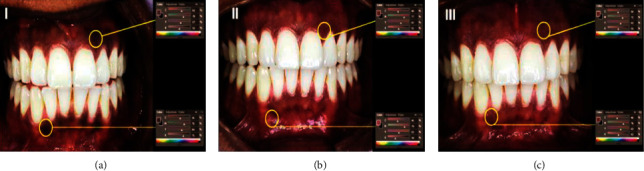
Assessment of gingival pigmentation using Photoshop software. (a) Preoperative image, (b) one month after operation, and (c) three months after operation.

The gingival ablation of each segment of the jaws extending from the central incisor to the second premolar was assessed before the treatment (preoperatively) and after the treatment (postoperatively) at two weeks, one month, three months, and six months. The intensity of gingival pigmentation was assessed using Dummett–Gupta oral pigmentation (DOP) index [14] and Hedin melanin index (HMI) [15]. HMI was used to define the extent of the pigmented areas [26] (Table 2). Clinical photographs were captured using a single digital camera (Samsung A 5- Korea) with standardization (resolution, 16 megapixels; distance, 15 cm from the pigmented area with fixed magnification) for all patients on recall visits. Photoshop software (Adobe system, United States) was used to analyze the pre- and postoperative photographs (Figure 1). Before the clinical photographing, one of the authors was trained by an expert on how to use the software and trace and measure the pigmented areas.

The hemostasis effect was evaluated by visual examination and based on the amount of bleeding noticed during the surgical procedure using bleeding index (BI) (Table 3), while the wound healing was assessed on the first day, one week and one month after the surgery [9] using the wound healing index (WHI) (Table 3).

All patients were called on the evening of the operation (day 1) and day 2 and day 3 postoperatively and asked to mark the level of the experienced pain based on a 0 to 10 numeric pain rating scale; with left end (0) point marked as “no pain” and the right end [10] marked as “severe pain.” A mark was placed at the corresponding number to match the level of the experienced pain [16]. The scores were calculated as shown in Table 4.

The data were analyzed using the GraphPad Prism 5 program (GraphPad Prism 5.01). The data were independently compared between the two groups (Er:YAG and diode laser groups) using paired *t*-test. The mean ± standard error of measurements (SEM) was used to identify the significant difference between the two groups. The difference is considered significant when the *p* value is less than 0.05 (*p* ≤ 0.05).

## 3. Results

**Figure 2:**
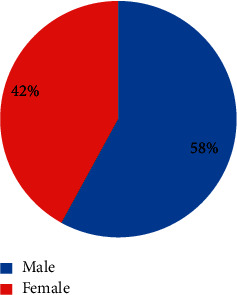
Gender distribution of the participants.

Out of the 15 recruited patients, 3 were dropped out as they had to travel. A total of 12 patients completed the treatment and followed for 6 months. The patients' age means were 28.6 ± 7.8 years, with males forming 58% of the participants (Figure 2). All of the subjects were recalled at one week, one month, three months, and 6 months. The laser ablation was performed for the 12 patients (24 arch sides for each laser type).

In all cases, the degree of pigmentation before the treatment (preoperative baseline) was almost the same between the two sites with an average DOPI: 2.04 ± 0.21 for Er:YAG and 2.20 ± 0.20 for diode laser (Table 5). Nine patients with 36 treated sites (75%) were free from any pigmentation after full treatment and by the end of the first month. However, three cases with 12 treated sites showed grade 1 pigmentation with both types of lasers at the first month with average DOPI of 0.26 ± 0.14 for Er:YAG and 0.25 ± 0.13 for diode treated sites (Table 5), although the difference was statically nonsignificant (*p*=0.9314).

Bleeding or haemorrhages were assessed at the time of the operation. The tendency for bleeding was noticed to be higher at ER:YAG laser-treated sites with an average BI score of 1.750 ± 0.13, while for diode laser-treated sites, it was 1.167 ± 0.11. This difference was statistically significant (paired *t*-test, *p* = 0.0027) (Table 6).

All cases showed complete healing (complete epithelialization) after one month (Table 7). However, in the first week, the healing seemed to be better at the diode laser-treated sites compared to Er:YAG, although the difference was statistically nonsignificant.

Our results showed that all patients admitted less painful experienced in the first, second, and third days with the diode treated sites than Er:YAG. The average pain score with the diode laser was 1.333 ± 0.39, 1.083 ± 0.41, and 0.7500 ± 0.27 for the first, second, and third postoperative days, respectively, while for the Er:YAG laser, it was 1.875 ± 0.47, 1.250 ± 0.40, and 1.083 ± 0.41 for the first, second, and third postoperative days, respectively. However, the differences were statically nonsignificant (Table 8). Three patients admitted using painkillers after treatment.

The summary of the questionnaires about patients' satisfaction demonstrated that all of the participants were pleased with the surgery of both lasers. Nevertheless, a few patients were annoyed by the popping sound of the Er:YAG laser during the surgery.

## 4. Discussion

GHP is not a disease or medical problem but remains a source of concern for many people that invites them to seek esthetic treatment [3]. Intraorally, gingivae followed by hard palate and buccal mucosae are the most common sites for physiological melanin pigmentation [5]. This is presumably attributed to the high number of melanocytes present in the gingiva compared to other parts of the oral mucosa [17].

Many techniques have been used for the treatment of gingival pigmentation; in spite of some disadvantages, the surgical approach remains the gold standard for GD [9]. Gingivectomy, for example, may be associated with alveolar bone loss leading to prolonged healing by secondary intension causing severe pain [11]. Despite the effectiveness of cryosurgery and electric surgery depigmentation [18], a highly skilled operator is required to manage the complicated technique and instrumentation. Gingival abrasion technique using a large round diamond bur in a high-speed handpiece with abundant irrigation has also been practiced [9]. The main problem with this technique is the difficulty in controlling the depth of the depigmentation and obtaining adequate access [19].

Recently, various types of laser have been used for gingival ablation, which are recognized as one of the most effective, comfortable, and reliable techniques with almost no postoperative complications [8, 10, 11, 20]. In this study, we compared the efficacy of diode laser (980 nm) and erbium-YAG laser (2940 nm) using the split-mouth technique for GD. The outcomes have shown that both lasers are efficient for gingival ablation.

One of the strongest points of this study is that neither local infiltration nor topical anesthesia was used, taking into consideration that laser ablation invariably causes trivial discomfort. In fact, it was our intention to prove that laser ablation is a painless procedure. The pain during the surgical procedure was controlled by lowering the hertz of the laser. This is a relatively novel technique and infrequently reported. The split-mouth technique has several advantages including the elimination of the confounding factors such as age, gender, and racial differences [21].

The current study demonstrated that both laser groups (Er:YAG and diode lasers) have nonsignificant differences in the GD efficacy. These findings are in line with the previous studies [22–25]. After one month from initiation of the treatment, all of the ablated sites of the cases were free of any pigmentation except three cases (2 of them were siblings) that showed grade 1 pigmentations with both types of lasers.

It has been noticed that the limited postablation repigmentation showed an increase in the areas ablated with Er:YAG-DOP 0.35 ± 0.18 and 0.39 ± 0.20 at three and six months, respectively, and with diode laser-DOP 0.32 ± 0.17 and 0.36 ± 0.19 at three and six months, respectively. At 3- and 6-month reevaluation revealed no repigmentation at the ablated sites. Although our results showed a higher rate of gingival pigmentation remnants than other studies, this could be attributed to the low laser parameters that were used by the author to reduce the pain. Nevertheless, these findings are still in agreement with the studies that used Er:YAG and diode laser for GD [10]. It is well known that diode laser wavelength is perfectly absorbed by haemoglobin and melanin pigments, while Er:YAG is absorbed by water. Also, Er:YAG has a shallow penetration, while diode laser has deep penetration that enables the removal of the deeply seated melanocytes in the gingival tissues [22].

In this study, the patients experienced slightly more pain at the Er:YAG laser-treated sites compared to the diode laser-treated sites. This could be attributed to the shallow surface interaction of the Er:YAG laser beam that requires more time to remove the pigments causing damage to the gingival tissues and subsequent postoperative pain. However, in this study, the pain was always controlled by using a lower power setting. Simsek Kaya et al. (2012) [26] experienced a similar clinical finding, a slight pain with the use of ER:YAG (score 1.0), and recommended using a lower power setting and multiple sessions. In the current study, the authors had to change the setting of the Er:YAG by increasing the hertz that resulted in a decrease in the overall energy to reduce the pain. This setting protocol is in line with a recent study by Gholami et al. (2018) [27], in which the authors demonstrated that by changing the Er:YAG laser setting, the pain can be controlled during surgery and postoperatively as well.

Our results revealed that the bleeding was significantly higher in the ER:YAG laser-treated sites with an average BI score of 1.750 ± 0.1306, while it was 1.167 ± 0.1124 (*P* ≤ 0.05) for diode. This finding is in agreement with Giannelli et al. (2014) study [28]. The better hemostatic effect of the diode laser is ascribed to the ability of its wavelength to deeply penetrate the soft tissues compared to the Er:YAG wavelength. Also, the diode laser is not absorbed by water; consequently, more heat is generated, which occludes the blood vessels, while the Er:YAG is absorbed by water and generates less heat [22]

The healing process was satisfactory for both lasers, exhibiting complete epithelialization after one month. No wound complications such as scars, ulcers, or infections were noticed. These observations are well documented in the literature confirming the uneventful gingival wounds after laser ablation [24, 26–28]. The former could be attributed to the bactericidal effect of the laser as a result of the generation of the free reactive oxygen radicals in the irradiated tissues leading to the sterilization of the surgical field. Furthermore, emission of low-level laser (LLL) from Er:YAG results in further stimulation of fibroblasts to release collagen fibers and extracellular matrices [29]. However, in the first week, the diode laser tended to accelerate healing compared to the Er:YAG, although the difference was statistically nonsignificant. The diode laser is considered superb for soft tissue surgery and shows no interaction with the hard dental tissues. Also, it has a thermal effect that results in the production of a thick coagulation layer on the treated surfaces that enhances proper healing [10]. On the other hand, Er:YAG laser, due to its specific wavelength, is highly absorbed by water resulting in less heat generation and better wound healing. Nevertheless, it interacts with the heart tissues. The reason is considered inferior to the diode laser when wound healing is considered. In addition, the setting of Er:YAG laser is crucial, as it can greatly affect the treatment outcomes.

At the final reevaluation visits (at 6 months), gingival repigmentation was not observed among the treated cases. It is inevitable to remember that the authors followed the cases for a relatively short period time (6 months); thus repigmentation may appear at later stages. In fact, in Atsawasuwan and Nimmanon (2000) study, the gingival repigmentation was not observed until 12 months after GD using Nd:YAG laser [30]. Furthermore, Alhabashneh et al. (2018) [24] found that 30% of the patients had grade 1 pigmentation after six months from Er:YAG laser ablation.

Gholami et al. (2018) [27], compared two settings of Er:YAG laser during GD, and areas of repigmentation were reported at 3 months. This can be justified by the fact that melanin-producing cells (melanocytes) are deeply seated in the basal layer of tissues and Er:YAG laser has a shallow laser beam penetration distance compared to the diode laser [10, 23]. Therefore, some of the unablated cells are reactivated leading to melanin synthesis. Some authors postulated that the gingival repigmentation may be due to the potential migration of melanocytes from the untreated neighbouring areas [8]. We, the authors of this study, strongly support the latter theory, particularly those melanocytes present in the interdental papilla or free gingiva. In this clinical study, we have observed that most of the remained deep areas of pigmentations were in the interdental papillae. This is because of the difficulty in completely removing the pigments from the interdental papilla and to the increased thickness of the gingival tissues at these sites [24, 31]. Therefore, we advocate another cycle of ablation procedure a year after the initial treatment to ablate any recurrent pigmented spots.

## 5. Conclusion

The results of this study highlight the effectiveness of dental lasers, particularly the diode laser for GD. It shows very promising results in comparison to Er:YAG laser, when postoperative pain, bleeding, and pigmentation recurrence are considered. Nonetheless, Er:YAG laser provides a better tissue healing cascade. Also, we confirm that laser ablation can be performed without the use of anesthesia provided the laser parameters are well controlled.

### 5.1. Limitation of the Study

This study has several limitations. The sample size was relatively small, and this is due to the limited time given by the university, as the study was part of a master degree. Also, the quality of the clinical photos could have been improved by using a better camera, which was not available due to limited resources. However, the study is encouraging for further studies, particularly when anaesthesia free gingival ablation is considered.

## Figures and Tables

**Table 1:** Er:YAG 2940 nm and diode 980 nm laser parameters and specifications.

Specification	Laser type	
	Er:YAG 2940 nm	Diode 980 nm
Laser manufacturer	LAMBDA Dr. Smile	LAMBDA Dr. Smile
Model	Pulser	Wiser
Delivery system	Noncontact tip	Optical fiber
Emission mode	Free running pulse	Continuous wave
Energy distribution	Gaussian	Gaussian
Pulse width	100 *μ*s	20 *μ*s
Energy per pulse	100 mJ	30.0 J (total energy)
Pulse repetition rate	20 pps	8 times/Selting (initiation technique)
Tip diameter	800 *μ*m	400 *μ*m (fiber)
Tip-to-tissue	1 mm	5 mm
Tip area	0.0050 cm^2^	0.0013 cm^2^
Spot diameter at tissue	0.1251 cm	0.0400 cm
Spot area at tissue	0.0123 cm^2^	0.0013
Beam divergence	12.7 degrees	12.7 degrees
Water	1.5 ml/min	None
Air	None	None
Power range	2 watts	0.80–1.10 watts
Length of treatment	600 sec	240–600 sec
Speed of movement	1 mm/sec	2–10 mm/sec
Peak power	1000 watts	1.10 watts
Peak power density	81,394 w/cm	1,132 w/cm^2^
Average power density	163 w/cm^2^	637–875 w/cm
Total energy	1200 joules	480.0–264.0 joules
Energy density with movement	160 J/cm^2^	20.0–137,4 J/cm^2^

**Table 2:** Indices used for evaluation of the gingival pigmentation.

Grade	Degree of pigmentation index (DOPI)	Grade	Melanin index (Hedin)
0	No clinical pigmentation (pink-colored gingiva)	0	No pigmentation
1	Mild clinical pigmentation (mild light brown color)	1	One or two solitary unit(s) of pigmentation in papillary gingiva without the formation of a continuous ribbon between solitary units
2	Moderate clinical pigmentation (deep brown to black).	2	More than three units of pigmentation in papillary gingiva without the formation of a continuous ribbon
3	Severe clinical pigmentation (mixed color)	3	One or more short continuous ribbons of pigmentation
		4	One continuous ribbon including the entire area between canines

**Table 3:** Indices used for evaluation of bleeding and wound healing.

Grade	Bleeding index (BI)	Grade	Wound healing index (WHI)
1	No bleeding, complete homeostasis	1	Tissue necrosis
2	Isolated bleeding points during surgery (mild)	2	Ulcer formation
3	Moderate bleeding, but clear field	3	Incomplete epithelialization
4	Severe bleeding, difficulty in procedure	4	Complete epithelialization

**Table 4:** Indices used for evaluation of postoperative pain.

Grade	Pain index
0	No pain
1–3	Mild pain
4–6	Moderate pain
7–10	Severe pain

**Table 5:** Degree of pigmentation index (DOPI) of Er:YAG compared to diode lasers.

Measured parameter	Laser	Baseline	One month	Three months	Six months
DOPI	Er:YAG	2.04 ± 0.21	0.26 ± 0.14	0.35 ± 0.18	0.39 ± 0.20
	Diode	2.20 ± 0.20	0.25 ± 0.13	0.32 ± 0.17	0.36 ± 0.19
	*p* value	0.5853	0.9314	0.6612	0.9299

*p* ≤ 0.05 is considered significant.

**Table 6:** Bleeding index of diode compared to Er:YAG lasers.

Measured parameter	Laser	Bleeding index during surgery	*p* value
Bleeding	Er:YAG	1.750 ± 0.1306	0.0027
	Diode	1.167 ± 0.1124	

*p* ≤ 0.05 is considered significant.

**Table 7:** Wound healing of Er:YAG compared to diode lasers.

Measured parameter	Laser	1 day	1 week	1 month
	Er:YAG	0.9667 ± 0.01	3.167 ± 0.16	4
	Diode	0.9333 ± 0.07	3.500 ± 0.19	4
	*p* value	0.6789	0.2068	

*p* ≤ 0.05 is considered significant.

**Table 8:** Postoperative pain of Er:YAG compared to diode lasers.

Measured parameter	Laser	First postoperative day	Second postoperative day	Third postoperative pain
Pain	Er:YAG	1.875 ± 0.47	1.250 ± 0.40	1.083 ± 0.41
	Diode	1.333 ± 0.39	1.083 ± 0.41	0.7500 ± 0.27
	*p* value	0.3917	0.7772	0.5130

*p* ≤ 0.05 is considered significant.

## Data Availability

The clinical and statistical data used to support the findings of this study are available from the corresponding author upon request.
